# Impact of Perioperative Acetylsalicylic Acid (ASA) Administration on Postoperative Intracranial Hemorrhage (pICH) and Thromboembolic Events in Patients with Intracranial Meningiomas

**DOI:** 10.3390/jcm13154523

**Published:** 2024-08-02

**Authors:** Anatoli Pinchuk, Nikolay Tonchev, Klaus Peter Stein, Vanessa M. Swiatek, Claudia A. Dumitru, Belal Neyazi, Ibrahim Erol Sandalcioglu, Ali Rashidi

**Affiliations:** Department of Neurosurgery, Otto-von-Guericke-University Magdeburg, 39120 Magdeburg, Germany; anatoli.pinchuk@med.ovgu.de (A.P.); nikolay.tonchev@med.ovgu.de (N.T.); klaus-peter.stein@med.ovgu.de (K.P.S.); vanessa.swiatek@med.ovgu.de (V.M.S.); claudia.dumitru@med.ovgu.de (C.A.D.); belal.neyazi@med.ovgu.de (B.N.); erol.sandalcioglu@med.ovgu.de (I.E.S.)

**Keywords:** ASA, meningioma, craniotomy, hemorrhage

## Abstract

**Background**: In routine medical practice, patients are increasingly using ASA for primary and secondary prevention. Although many of these patients discontinue ASA prior to elective intracranial surgery, there are limited data to support whether perioperative ASA use raises the risk of postoperative hemorrhage. This study aimed to investigate the implications of continuing or stopping ASA around the time of surgery in patients with intracranial meningiomas, focusing on postoperative hemorrhage and thromboembolic events. **Methods**: For this purpose, medical records and radiological images of 1862 patients who underwent cranial neurosurgical procedures for brain tumors over a decade at our neurosurgical institute were retrospectively analyzed. The risk of postoperative hemorrhage was evaluated by comparing meningioma patients who received ASA treatment with those who did not. Furthermore, we investigated other factors that influence postoperative hemorrhage and thromboembolic events, particularly in patients receiving ASA treatment. **Results**: A total of 422 patients diagnosed with meningiomas underwent surgical intervention. Among the patients who received ASA preoperatively, 4 out of 46 (8.69%) experienced postoperative hemorrhage requiring surgical intervention, whereas the same complication occurred in only 4 out of 376 patients (1.06%) in the non-ASA group (*p* = 0.007). There was no significant difference in the incidence of thromboembolic events between the two groups. **Conclusions**: Our analysis revealed an increased risk of postoperative hemorrhage in patients using ASA.

## 1. Introduction

Meningiomas are present in up to 30% of all intracranial tumors [1,2,3]. Surgical resection is the preferred treatment for patients with symptomatic or growing meningiomas. Despite the curative intent of surgery and neurological improvement observed in most cases, operative interventions carry inherent risks [2,4], including postoperative hemorrhage [1], which can have devastating consequences for patients [5].

The use of antiplatelet agents, such as acetylsalicylic acid (ASA) for primary or secondary prophylaxis of cardiovascular conditions, poses a therapeutic challenge in neurosurgical patients, particularly in the elderly [6,7]. Low-dose ASA (50–100 mg/day) induces effective cyclooxygenase (COX)-1 inhibition and impairs thromboxane (Tx) A2 synthesis one hour after ingestion [7]. The irreversible effect of ASA persists for 8 to 10 days, lasting as long as platelets survive after leaving the bone marrow [8,9]. Maintaining antiplatelet agents during surgery may heighten the potential for hemorrhagic complications [10,11]. The devastating and potentially lethal consequences of hemorrhagic complications linked with a craniotomy are well documented [10,12]. The decision to discontinue ASA, especially in patients with a history of cardiovascular disease and stroke, presents a dilemma due to the increased likelihood of adverse events. Given the high morbidity, the risk of thromboembolic events might be elevated in this patient group. However, the influence of ASA usage on the risk of hemorrhage in meningiomas undergoing surgical resection, which already carry an elevated risk of hemorrhage, has seldom been investigated. The existing neurosurgical literature on cranial and spinal procedures typically does not indicate an increased risk of postoperative hemorrhage [13,14,15]. However, these studies often lack specificity regarding tumor characteristics or surgical aspects.

The incidence of venous thromboembolism (VTE) and bleeding immediately following tumor resection in patients with symptomatic benign tumors, such as meningiomas, remains poorly understood. Patients with intracranial metastases and gliomas commonly experience venous thromboembolism (VTE) during the course of their illness [16,17]. Mechanisms such as increased local synthesis of tissue factors, impaired postoperative mobility, hemiparesis, and genetic predisposition have been suggested as potential contributors to VTE development in brain tumor patients [18,19,20,21].

Due to pre-existing conditions like cardiovascular disease and stroke, the patients in our study were treated with ASA. We examined how these pre-existing conditions influenced the increased risk of developing thromboembolic events after surgery.

## 2. Methods

**Figure 1:**
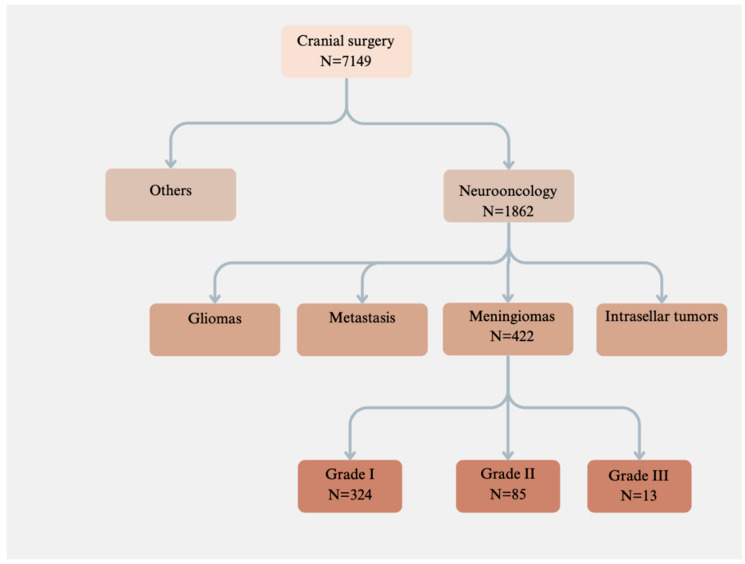
Between 2008 and 2018, our neurosurgery department performed 7149 cranial operations, including 422 meningioma surgeries.

A retrospective analysis was performed on the medical records and radiological images of 422 patients who had primary or recurrent meningioma operations at our institution from 2008 to 2018 (Figure 1).

Patients’ clinical data were collected through retrospective chart review, including demographic data (age, sex, blood group), clinical history, body mass index (BMI), perioperative administration of ASA treatment, hypertension, diabetes, smoking history, cardiovascular diseases, renal disease, chronic inflammation, operational and hospitalization data, recurrent operations (secondary meningioma operation), laboratory parameters, duration of hospitalization, operative procedure, duration of surgery, blood loss, and postoperative complications during the hospital stay. During the operation, the amount of blood suctioned was collected in a container. Postoperatively, the anesthesiologist and operating room staff measured the amount of fluid in the container and subtracted it from the total fluid administered for irrigation. This method enabled the measurement of intraoperative blood loss. Postoperative complications were classified according to Ibanez et al. [2,22,23] as follows:

- Grade I comprised non-life-threatening abnormalities from the usual postoperative course treated without invasive procedures;
- Grade II complications required invasive interventions such as surgical, endoscopic, and endovascular procedures;
- Grade III complications were life-threatening adverse events necessitating treatment in an intensive care unit, categorized into IIIa for complications with single organ malfunction and IIIb for complications with multiple organ malfunction;
- Grade IV complications included deaths resulting from complications.

In the absence of contraindications, pre- and postoperative imaging was performed by contrast-enhanced MRI. Steroids were administered preoperatively in case of tumor edema or space-occupying effects of the tumor. The choice of neurosurgical approach during tumor resection was made by the surgeon. Intraoperative ultrasound and electrophysiologic monitoring were available during the procedure, and frameless neuronavigation was available if necessary.

Tumor characteristics included the following:

- Tumor size;
- Localization (supra- or infratentorial, olfactory/sella/planum/falcine/parasagittal, convexity, sphenoid wing, other);
- Histopathological grading;
- Recurrence operation;
- Surgical resection according to the Simpson classification.

Operative parameters included blood loss during the surgery, duration of the surgery, extent of resection, and other characteristics. The Karnofsky Performance Scale (KPS) was assessed at admission before surgery and at discharge after hospitalization. The Glasgow Outcome Scale was used postoperatively to assess patient status at discharge.

Exclusion criteria were as follows:

- Age <18 years, pregnancy;
- Patients receiving other antiplatelet agents such as Clopidogrel and/or anticoagulation such as Marcumar.

To statistically assess the potential influence of ASA on postoperative hemorrhage, we used Fisher’s exact test. Patients were categorized into two groups:

- No ASA impact: patients with no history of ASA usage and/or discontinued ASA use (≥7 days prior to surgery);
- ASA impact: patients who continued ASA intake (intake ceased <7 days prior to surgery or not ceased at all).

### Intracranial Hemorrhage

All postoperative radiological findings were analyzed for the presence of hemorrhage. Additionally, radiological images of all intracranial hemorrhages were corroborated by two neurosurgeons to confirm their presence.

Hemorrhages were classified into the following categories:

- Hemorrhage in the tumor cavity;
- Intracerebral hemorrhage;
- Subarachnoid hemorrhage;
- Subdural hemorrhage.

Only patients who experienced neurological deterioration due to space-occupying hemorrhage requiring surgery were classified as having a significant postoperative hemorrhage. Neurological deterioration was defined as the presence of a focal neurological deficit, alteration in consciousness, or signs of increased intracranial pressure, such as headaches, nausea, or changes in cognitive function.

## 3. Statistical Analysis

Categorical variables were presented as counts (percentages), while continuous variables were expressed as medians with interquartile ranges (IQRs), given that all continuous variables in this study were non-normally distributed. This non-normal distribution was confirmed by the Kolmogorov–Smirnov test. The impact of categorical variables on pICH was analyzed using the χ2 test or Fisher’s exact test when cell counts were less than five. Differences in continuous variables between patients with and without pICH were assessed using the Wilcoxon Mann–Whitney-test. Variables that were significant in the univariate analysis were included as covariates in the multivariate analysis, which was conducted using logistic regression. For variables that showed substantial deviation from a normal distribution, a logarithmic transformation was applied. All statistical analyses were performed using the SAS University Edition software package 9.4 (SAS Institute, Inc., Cary, NC, USA) and SPSS for Windows version 18.0 (SPSS, Inc., Chicago, IL, USA). Two-sided *p* values ≤ 0.05 were considered statistically significant.

## 4. Results

### 4.1. Incidence of Intracranial Hemorrhage

A total of 422 patients received surgery for meningiomas during the specified period. Postoperatively, 40 patients developed a hemorrhage, with only 8 patients requiring reoperation due to neurological deterioration or space-occupying hemorrhage. In the No ASA impact group, 33 patients had postoperative hemorrhage, and 4 (12%) of them underwent surgical revision.

**Figure 2:**
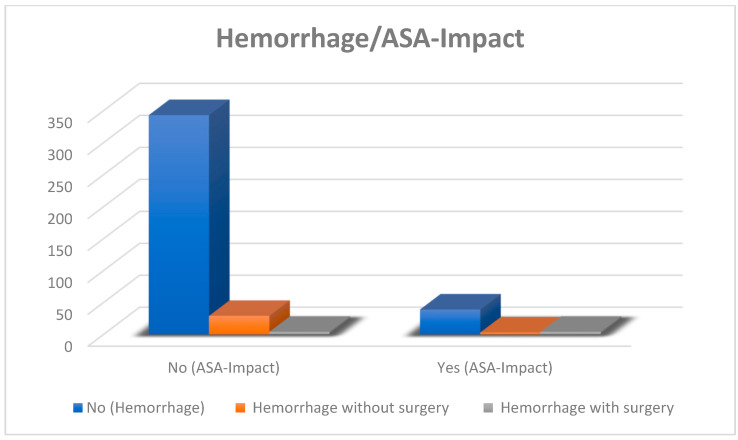
Of 422 surgical patients, 40 (9%) in both the ASA and No ASA impact groups had postoperative hemorrhage. In the ASA group, 4 out of 7 (57%) required revision surgery; in the No ASA impact group, 4 out of 33 (12%) required surgery.

A total of 71 patients (16.8%) had a history of ASA use at the time of surgery. Of the ASA users, 46 patients (10.9%) discontinued ASA for less than 7 days or not at all (ASA impact). Seven patients from the ASA impact group experienced postoperative hemorrhage, and four of them necessitated revision surgery (Figure 2). Additionally, 7 out of 46 patients (15%) in the ASA impact group and 33 out of 376 patients (8%) in the No ASA impact group experienced a hemorrhage, with or without surgery. The risk of hemorrhage was higher in the ASA group (*p* = 0.011).

### 4.2. Intracranial Hemorrhage Relative to ASA Intake

**Figure 3:**
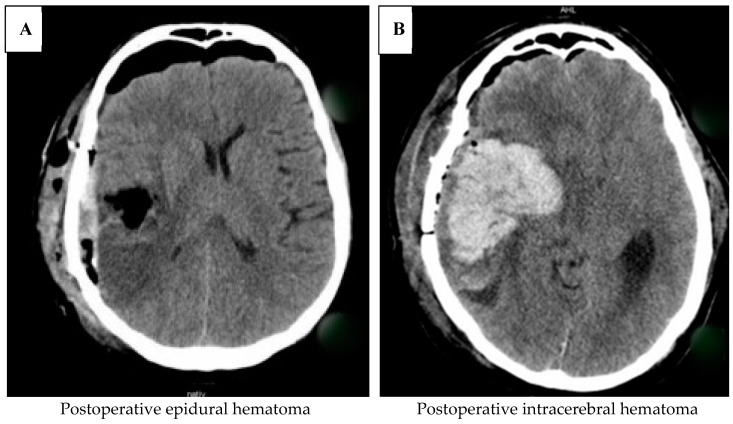
Examples of patients’ hemorrhage during postoperative CT checks. (**A**) shows a subdural hemorrhage without the requirement for surgery, whereas (**B**) illustrates a severe intracerebral hemorrhage that made reoperation necessary.

Table 1 displays patients in the ASA and No ASA impact groups without postoperative hemorrhage, as well as those requiring surgical intervention due to postoperative hemorrhage. Patients who experienced postoperative bleeding but did not require surgery are not included in the list. Continued use of ASA was associated with a significantly higher incidence of postoperative hemorrhage requiring reoperation (*p* = 0.007). Figure 3 shows two patients with post-operative hemorrhage.

### 4.3. Hemorrhage, Demographic Data, Tumor Characteristics, and Laboratory Parameters

Demographic characteristics and additional patient data including sex (*p* = 0.637), blood group (*p* = 0.624), smoking status (*p* = 0.860), age at surgery (*p* = 0.620), BMI (*p* = 0.603), and comorbidities such as diabetes (*p* = 0.214), cardiovascular disease (*p* = 0.681), hypertension (*p* = 0.293), dyslipoproteinemia (*p* = 0.609), renal disease (*p* = 0.764), liver disease (*p* = 0.683), and chronic inflammation (*p* = 0.615) did not correlate with postoperative hemorrhage. Intraoperative parameters, including duration of surgery (*p* = 0.649) and blood loss during the operation, were also evaluated. Blood loss during surgery was found to have a significant and direct influence on the risk of postoperative hemorrhage (*p* = 0.045). The Karnofsky Performance Scale (KPS) and Glasgow Outcome Scale (GOS) scores of patients with hemorrhage were significantly worse after surgery (*p* = 0.001). In addition, these patients tended to endure a prolonged hospital stay (Mann–Whitney U test, exact significance: two-sided, 0.097, one-sided, 0.048). Table 2 shows the important demographic, comorbidities, and intra- and postoperative characteristics.

Table 3 shows the tumor characteristics and laboratory parameters. Preoperatively assessed laboratory parameters, particularly coagulation factors, did not significantly influence the risk of postoperative hemorrhage.

Tumor characteristics such as histopathological grade (*p* = 0.399), recurrent meningiomas (*p* = 0.604), location of the tumor (*p* = 0.288), tumor site (*p* = 0.500), and resection grade according to Simpson classification (*p* = 0.564) also had no correlation with postoperative hemorrhage either.

Table 4 refers to patients who received preoperative ASA and demonstrates which factors are significantly different among patients taking ASA compared to those not taking ASA. In contrast, Table 3 illustrates the significant factors leading to hemorrhage. Patients treated with ASA tend to be male and have pre-existing conditions such as diabetes, cardiovascular disease, and hypertension. In addition, patients over 62.5 years were more likely to use ASA (*p* = 0.001).

### 4.4. Complications According to Ibanez Classification

The surgical complications, such as cerebrospinal fluid leaks, wound infection and hemorrhage, have been categorized according to the Ibanez classification and are listed in Table 5. The classification was introduced in the methodology section. The frequency of surgical complications, categorized using the Ibanez classification, did not differ significantly between the ASA and No ASA impact groups (*p* = 0.567).

### 4.5. VTE and ASA Intake

Of the 422 patients, 8 (1.9%) developed postoperative deep vein thrombosis (DVT) during their hospital stay. It is noteworthy that postoperative DVT occurred in six patients (1.6%) who were in the No ASA impact group. A total of 71 patients had a history of ASA use. Intriguingly, DVT manifested more than twice as often in the subgroup that continued ASA intake compared to those who discontinued ASA use before surgery (*p* = 0.213). Symptomatic pulmonary embolism occurred in eight patients (2.1%) without ASA use, while no occurrences were observed in the ASA impact group (*p* = 0.630). Furthermore, the observed numbers lacked statistical significance, likely attributable to the small sample size. All patients received compression stockings after the operation and were mobilized starting the day after surgery. Even patients with paralysis, who could not be fully mobilized, received professional physiotherapy with movement exercises in bed postoperatively.

## 5. Discussion

Preoperative ASA therapy poses a dilemma for neurosurgeons [7,24]. While ASA administration is imperative for managing various disorders [6], the risk of postoperative hemorrhage presents dire consequences for patients [11]. Several studies, including those by Hanalioglu et al., did not find an increased risk of hemorrhage associated with cranial surgery [10,13,25,26]. However, these studies did not differentiate between different types of cranial surgeries, leaving uncertainty regarding the generalizability of these findings to all cranial surgeries and tumors with varying characteristics. Similar results to those aforementioned studies were observed in lumbar neurosurgical operations and specific cranial surgeries, wherein continuous ASA application showed no significant adverse effects [14,15]. In this study, our approach steers away from drawing broad conclusions across various neurosurgical procedures. Instead, we focus on assessing the impact of ASA administration on specific tumors. In addition to analyzing general operative characteristics and postoperative complications, we conducted a retrospective evaluation to assess the impact of continuous ASA use in patients undergoing surgery for meningiomas over a period exceeding 10 years. Contrary to the previous results [5], we found a significantly higher risk of postoperative hemorrhage in patients continuing ASA intake. Moreover, the risk of hemorrhage was notably reduced in patients who discontinued ASA therapy more than 7 days prior to surgery.

Consistent with previous research, we observed higher intraoperative blood loss in patients receiving continuous ASA therapy, which also aligns with the results of Rahman et al. [10] in cranial surgery and other neurosurgical studies in the spinal lumbar setting [14,27,28]. Despite the growing utilization of ASA for primary and secondary prevention, particularly among the elderly population, our findings reaffirm the detrimental effects of postoperative hemorrhage with a cutoff age of 62.5 in our study, which are associated with worse outcomes and prolonged hospital stays [6,7].

Efforts should be prioritized to achieve total resection of intracranial tumors, aiming to minimize the risk of postoperative hematomas [29,30]. Such tumors often present with a substantial mass that has exerted prolonged compression on and deformation of the brain parenchyma, defining characteristic features of these scenarios. Consequently, the local elasticity of the brain diminishes over time. After tumor removal, a resection cavity often remains, which may gradually accumulate blood over the following hours or days due to minor capillary and/or venous bleeding [29,30,31,32].

We could not find any correlation between tumor characteristics such as tumor grade, tumor recurrence, localization, especially the degree of resection—classified according to Simpson—and bleeding risk after surgery.

It has been pointed out that the duration of brain tumor surgery is an independent risk factor for extracranial complications and that the potential harm of slow surgery should be of interest to neurosurgeons [31]. The duration of surgery, along with comorbidity and acquired neurological deficits, is an independent risk factor for extracranial complications after brain tumor surgery [31]. However, the general surgical risk can be minimized by shortening the duration of surgery. In 2018, Zheng et al. showed that early defect coverage is associated with a shorter duration of surgery so that a better outcome for the patient can be expected [29]. In our study, no significant association was found between the duration of surgery and the risk of hemorrhage. Although, there was a trend. It is important to mention that the operation is performed by experienced and sometimes less experienced colleagues, which is why the duration of the operation varies and the results are not entirely reliable.

The degree of vascularization of operating lesions may influence the development of postoperative intracranial hemorrhage. Kalfas and Little observed that tumors, particularly meningiomas, were associated with the highest incidence of secondary bleeding in intracranial surgeries [33]. This finding is supported by similar reports from other researchers. The pathomechanisms underlying the elevated risk of secondary hemorrhage in hypervascularized processes such as meningiomas may involve pathological vessel walls and the activity of tumor-specific enzymes and growth factors [33,34]. Although a platelet count below 150/mL has been linked to an increased risk of pICH [35], this association was not statistically confirmed in our study due to the limited number of patients with thrombocytopenia. In a small number of patients with pICH and pathologic PTT, INR, or Quick values, statistical calculation regarding pICH was not presentable. Several pertinent risk factors for pICH, including preoperative and early postoperative hypertensive crises [36,37,38], vasculopathy-associated diabetes mellitus [39], cerebral amyloid angiopathy [40], factor XIII deficiency [1], or alcohol abuse [41], have been identified by other researchers but were not specifically investigated in this study. Nonetheless, these factors represent crucial observations highlighted by various authors and warrant consideration in future studies. Specifically, the patients with pICH experienced a significant deterioration in both Glasgow Outcome Scale (GOS) and Karnofsky Performance Scale (KPS) scores after surgery. Such outcomes underscore the devastating consequences of postoperative bleeding in affected patients.

If we categorize all identifiable risk factors for pICH as controllable or uncontrollable, a comprehensive approach to patient management emerges. Controllable factors, including ASA use, coagulation disorders such as thrombocytopenia and factor XIII deficiency, significant intraoperative blood loss, and pre- and perioperative hypertensive crises, offer avenues for intervention. By stabilizing these modifiable factors, the incidence of pICH can be reduced, subsequently lowering morbidity and mortality rates. Conversely, uncontrollable factors such as age, underlying pathology, tumor location, cerebral amyloid angiopathy, and vasculopathy in diabetes mellitus represent inherent challenges that must be carefully considered in patient care strategies.

### 5.1. VAE and ASA

Deep vein thrombosis/pulmonary embolism (DVT/PE) represents a significant complication in neurosurgical patients, contributing to increased morbidity, mortality, prolonged hospitalization, and elevated hospital costs [42]. Patients with meningiomas are known to face an elevated risk of DVT [30,32,43,44]. Additionally, active cancer has been identified as a strong risk factor for DVT complications [45]. Several mechanisms may play a role, including tumor-induced hypercoagulable states and increased adhesion and platelet aggregation [46]. Previous studies have reported results on the correlation between WHO grade and the incidence of DVT/PE [32,44].

We did not find a correlation between preoperative ASA use and postoperative thrombosis in meningioma surgery. However, it is not excluded that we may have missed such an association due to the small sample size. Thrombosis occurred more than twice as frequently in the subgroup that continued to take ASA than in the group that discontinued ASA before surgery. Although this finding is not statistically significant due to the small sample size, it suggests that patients in the group taking ASA may have had underlying morbidity.

In the absence of prospective screening for deep vein thrombosis and pulmonary embolism, which may be clinically unremarkable, it is likely that the full extent of the thromboembolic adverse events is underestimated because asymptomatic patients were inadvertently excluded.

Our analysis showed that patients who took ASA before removing hypervascularized intracerebral meningiomas were at risk of experiencing pICH. Therefore, it is recommended to discontinue ASA preoperatively for elective meningioma surgeries. However, our study has certain limitations of a retrospective analysis. We also discussed some factors influencing the occurrence of pICH.

### 5.2. Limitation

Our study has the limitation of being a retrospective study. In addition, prospective studies should validate the results with multiple tests and thrombocyte function tests in patients treated with ASA preoperatively. Some patients are also ASA non-responders and should be excluded from the study after preoperative testing.

## Figures and Tables

**Table 1:** Four patients in the ASA impact group and four patients in the No ASA impact group experienced postoperative hemorrhage requiring surgical intervention. Patients treated with ASA had a significantly increased risk of postoperative hemorrhage (*p* 0.007).

				Hemorrhage with Surgery	No Hemorrhage	*p*-Value
				N (%)	N (%)	
ASA Impact	Yes No		∑			0.007
			43 (11.0)	4 (50.0)	39 (10.2)	
			347 (89.0)	4 (50.0)	343 (89.8)	
		∑ (%)	390	8 (100.0)	358 (100.0)	

**Table 2:** Comparing demographic data, comorbidities, intra- and postoperative characteristics, only GOS and KPS exhibited a significant difference between patients with and without hemorrhage (exclusion of cases with bleeding without surgery), Glasgow Outcome Scale (GOS), Karnofsky Performance Scale (KPS).

	Parameters		Hemorrhage		
			No N 382 (%) Mean # SD	Yes with Surgery N 8 (%), Mean # SD	*p*-Value
Demographic data	Sex	Female	257 (67.3)	6 (75.5)	0.637
		Male	125 (32.7)	2 (25.5)	
	Age		61.7	64.1	0.620
	BMI		28.5	27.5	0.603
	ASA classification	I	27 (7.1)	0 (0.0)	0.859
		II	236 (61.9)	5 (62.5)	
		III–IV	118 (31.1)	3 (37.5)	
	Smoker	Yes	84 (23.3)	2 (25.5)	0.860
		No	292 (77.7)	6 (75.5)	
Intra-and postoperative factors	Duration of the operation, [min.]		234.2	271.2	0.649
	Blood loss [mL]		579.2	1143.7	0.045
	Duration of stay, (day)		13.41	15.9	0.097
	GCS		4.5	3.1	<0.001
	KPS		76	50	<0.001
Comorbidities	Hypertension	Yes	202 (52.9)	6 (75.0)	0.293
		No	180 (47.1)	2 (25.0)	
	Diabetes	Type I/II	81 (21.2)	0 (0.0)	0.214
		No	301 (78.8)	8 (100.0)	
	Coagulopathy	Yes	5 (1.3)	1 (12.5)	0.118
		No	377 (98.7)	7 (87.5)	
	Cardiovascular diseases	Yes	56 (14.7)	1 (12.5)	0.681
		No	326 (85.3)	7 (87.5)	
	Chronic inflammation	Yes	38 (9.9)	0 (0.0)	0.615
		No	344 (90.1)	8 (100.0)	

**Table 3:** No significant differences were found between patients with and without hemorrhage when analyzing preoperative laboratory parameters and tumor characteristics, Gpt, and giga particle.

	Parameters		Hemorrhage		
			No N 382 (%), Mean # SD	Bleeding with Surgery N 8(%), Mean # SD	*p*-Value
Laboratory Parameters	INR		0.98	1.05	0.627
	Platelets [10 × 9/L]		270.4	314.7	0.719
	C-reactive protein [mg/L]		8.4	3.9	0.572
	Leukocytes [Gpt/L]		8.7	7.9	0.704
Tumor characteristics (Meningiomas)	WHO Grade	I	286 (75.1)	8 (100.0)	0.399
		II	82 (21.5)	0 (0.0)	
		III	13 (3.4)	0 (0.0)	
	Recurrence	Yes	51 (13.4)	0 (0.0)	0.604
		No	331 (86.6)	8 (100.0)	
	Localization	frontal	110 (28.8)	2 (25.0)	0.288
		temporal	28 (7.3)	2 (25.0)	
		parietal	73 (19.1)	1 (12.5)	
		occipital	18 (4.7)	0 (0.0)	
		cerebellar	22 (5.8)	0 (0.0)	
		intra/suprasellar	6 (1.6)	1 (12.5)	
		skull base	113 (29.6)	2 (25.5)	
		CPA	12 (3.1)	0 (0.0)	
	Side	left	175 (45.8)	5 (62.5)	0.500
		middle	41 (10.7)	1 (12.5)	
		right	166 (43.5)	2 (25.0)	
	Simpson Grade	I	63 (16.5)	3 (37.5)	0.564
		II	160 (41.9)	3 (37.5)	
		III	87 (22.8)	1 (12.5)	
		IV	68 (17.8)	1 (12.5)	
		V	4 (1.0)	0 (0.0)	

**Table 4:** (a). Patients in the ASA impact group were older than those in the No ASA impact group, had a poorer ASA classification, and suffered more frequently from concomitant diseases such as high hypertension, diabetes, and cardiovascular diseases. Glasgow Outcome Scale (GOS), Karnofsky Performance Scale (KPS). The significant parameters are marked in bold. (b). Patients in the ASA impact group and in the No ASA impact group do not show any differences regarding laboratory parameters and tumor characteristics.

(a)					
	Parameters		ASA Preoperatively		
			No ASA Impact N 376(%), Mean #SD	ASA Impact N 46(%) Mean #SD	*p*-Value
Demographic data	Sex	Female	261(69.4)	25 (54.3)	**0.039**
		Male	115 (30.6)	21 (45.7)	
	Age		61.2	70.8	**<0.001**
	Height		168.0	167.6	0.756
	Weight		80.1	82.9	0.197
	ASA classification	I	20 (8.0)	0 (0.0)	**<0.001**
		II	243 (64.8)	19 (41.3)	
		III–IV	102 (27.2)	27 (58.7)	
	Smoker	Yes	82 (22.2)	9 (20.0)	0.741
		No	288 (78.8)	36 (80.0)	
Operational factors	Duration of the operation [min.]		239.3	232	0.627
	Blood loss [mL]		611.5	588.0	0.394
	Duration of Stay [Day]		13.8	12.1	0.342
	GCS		4.45	4.41	0.712
	KPS		74.6	73.9	0.945
Comorbidities	Hypertension	Yes	190 (55.5)	36 (78.3)	**<0.001**
		No	186 (49.5)	10 (21.7)	
	Diabetes	Type I/II	75 (19.9)	18 (39.1)	**0.003**
		No	301 (80.1)	28 (60.9)	
	Coagulopathy	Yes	6 (1.6)	1 (2.2)	0.557
		No	370 (98.4)	45 (97.8)	
	Cardiovascular	Yes	44 (11.7)	20 (43.5)	**<0.001**
		No	322 (88.3)	26 (56.5)	
		No	322 (88.3)	26 (56.5)	
	Chronic inflammation	Yes	40 (10.6)	4 (8.7)	0.804
		No	336 (89.4)	42 (91.3)	
**(b)**					
	**Parameters**		**ASA Preoperatively**		
			**No ASA Impact** **N 376(%), Mean #SD**	**ASA Impact** **N 46(%) Mean #SD**	***p*-Value**
Laboratory Parameters	INR		0.98	1.00	0.348
	Platelets [10 × 9/L]		269	275	0.690
	C-reactive protein [mg/L]		8.2	9.7	0.055
	Leukocytes [Gpt/L]		8.7	8.7	0.677
Tumor characteristics (Meningiomas)	WHO Grade	I	292 (77.9)	32 (69.6)	0.358
		II	72 (19.2)	12 (26.1)	
		III	11 (2.9)	2 (4.3)	
	Recurrence	Yes	47 (12.5)	7 (15.2)	0.603
		No	329 (87.5)	39 (84.8)	
	Localization	frontal	101 (62.9)	21 (45.7)	0.344
		temporal	29 (7.7)	3 (6.5)	
		parietal	71 (18.9)	7 (15.2)	
		occipital	16 (4.3)	2 (4.3)	
		cerebellar	22 (5.9)	1 (2.2)	
		intra/suprasellar	7 (1.9)	1 (2.2)	
		skull base	118 (31.4)	11 (23.9)	
		CPA	12 (3.2)	0 (0.0)	
	Side	left	172 (45.7)	20 (43.5)	0.685
		middle	41 (10.9)	7 (15.2)	
		right	163 (43.4)	19 (41.3)	
	Simpson Grade	I	62 (16.5)	13 (28.3)	0.379
		II	161 (42.8)	17 (37.0)	
		III	80 (21.3)	10 (21.7)	
		IV	69 (18.4)	6 (13.0)	
		V	4 (1.1)	0 (0.0)	

**Table 5:** Frequency of complications in ASA versus No ASA groups using the Ibanez classification. It can be shown that there is no relevant difference in either group (*p* = 0.567).

Surgical Complications according to Ibanez’s Classification × Hemorrhage					
			ASA Preoperative		Total
			No ASA Impact	ASA Impact	
Surgical complications	0	N (%)	265 (70.5%)	29 (63.0%)	294 (69.7%)
END	Ia/Ib	N (%)	63 (16.8%)	8 (17.4%)	71 (16.8%)
CFS leak/Wound infection	IIa/IIb	N (%)	22 (5.9%)	4 (8.7%)	26 (6.2%)
Hemorrhage	IIIa/IIIb	N (%)	19 (5.1%)	4 (8.7%)	23 (5.5%)
Death	IV	N (%)	7 (1.9%)	1 (2.2%)	8 (1.9%)
Total		N (%)	376 (100%)	46 (100%)	422 (100%)

Complications are classified as none, nonsevere (Ibanez I and II), or severe (Ibanez III and IV). CSF, cerebrospinal fluid, END, early neurological deterioration.

## Data Availability

The datasets obtained and analyzed during the current study are available from the corresponding author on reasonable request.
